# Coronary Large Conductance Ca^2+^-Activated K^+^ Channel Dysfunction in Diabetes Mellitus

**DOI:** 10.3389/fphys.2021.750618

**Published:** 2021-10-21

**Authors:** Tong Lu, Hon-Chi Lee

**Affiliations:** Department of Cardiovascular Medicine, Mayo Clinic, Rochester, MN, United States

**Keywords:** BK channel, diabetes mellitus, coronary arteries, blood vessels, regulation

## Abstract

Diabetes mellitus (DM) is an independent risk of macrovascular and microvascular complications, while cardiovascular diseases remain a leading cause of death in both men and women with diabetes. Large conductance Ca^2+^-activated K^+^ (BK) channels are abundantly expressed in arteries and are the key ionic determinant of vascular tone and organ perfusion. It is well established that the downregulation of vascular BK channel function with reduced BK channel protein expression and altered intrinsic BK channel biophysical properties is associated with diabetic vasculopathy. Recent efforts also showed that diabetes-associated changes in signaling pathways and transcriptional factors contribute to the downregulation of BK channel expression. This manuscript will review our current understandings on the molecular, physiological, and biophysical mechanisms that underlie coronary BK channelopathy in diabetes mellitus.

## Introduction

Diabetes mellitus (DM) has become a global epidemic. The incidence and the prevalence of DM have steadily increased over the past few decades. According to the WHO report in 2021, over 422 million people worldwide have DM with a prevalence of 8.6%, causing 1.6 million deaths annually.^1^ Type 1 diabetes mellitus (T1DM) accounts for 5–10% of the total cases of DM and is caused by autoimmune-mediated destruction of pancreatic β-cells, leading to hyperglycemia and insulin dependence (Bluestone et al., 2010; Op De Beeck and Eizirik, 2016). Type 2 diabetes mellitus (T2DM) represents 90–95% of the total cases of DM and is caused by insulin resistance with hyperinsulinemia, hyperglycemia, and hyperlipidemia in most patients (Pandey et al., 2015; Halim and Halim, 2019).

Both T1DM and T2DM are intimately related to micro-vascular and macro-vascular diseases, including ischemic heart disease, cerebrovascular disease, and peripheral vascular disease, resulting in myocardial infarction, stroke, retinopathy, nephropathy, and neuropathy with organ and tissue damages in 70% of diabetic patients (Kurisu et al., 2003; Yeung et al., 2012; Beckman and Creager, 2016; Sorop et al., 2016). The clinical consequences of diabetic vascular complication are devastating. DM is the leading cause of end stage renal disease, new cases of blindness, and non-traumatic lower extremity amputation, imposing global direct health expenditure of $ 760 in 2019 with a projected $ 825 billion by 2030 and $ 845 billion by 2045 (Williams et al., 2020). Hence, it is critically important to understand the mechanisms of vascular dysregulation in DM so that better diagnostic and therapeutic approaches can be developed to treat diabetic vascular complications more effectively.

Ionic mechanisms play a central role in the regulation of vascular reactivity. Vascular large conductance Ca^2+^-activated K^+^ (BK) channels are major determinants of such regulation. BK channels are densely populated in vascular smooth muscle cells (SMCs), particularly in small resistance arteries, and provide tight regulation of vascular tone and tissue perfusion. It is well established that vascular BK channel expression and function are abnormal in DM. Diabetic patients are known to have worse cardiovascular events and outcome, with higher risks of ischemic heart disease and myocardial infarction (Kurisu et al., 2003; Yeung et al., 2012; Sorop et al., 2016). In this review, we will focus on recent findings in the coronary arterial SMCs, highlighting the diabetes-mediated changes in channel expression, function, and intrinsic properties, as well as the molecular mechanisms associated with these changes.

## Structure and Function of Vascular BK Channels

**Figure 1:**
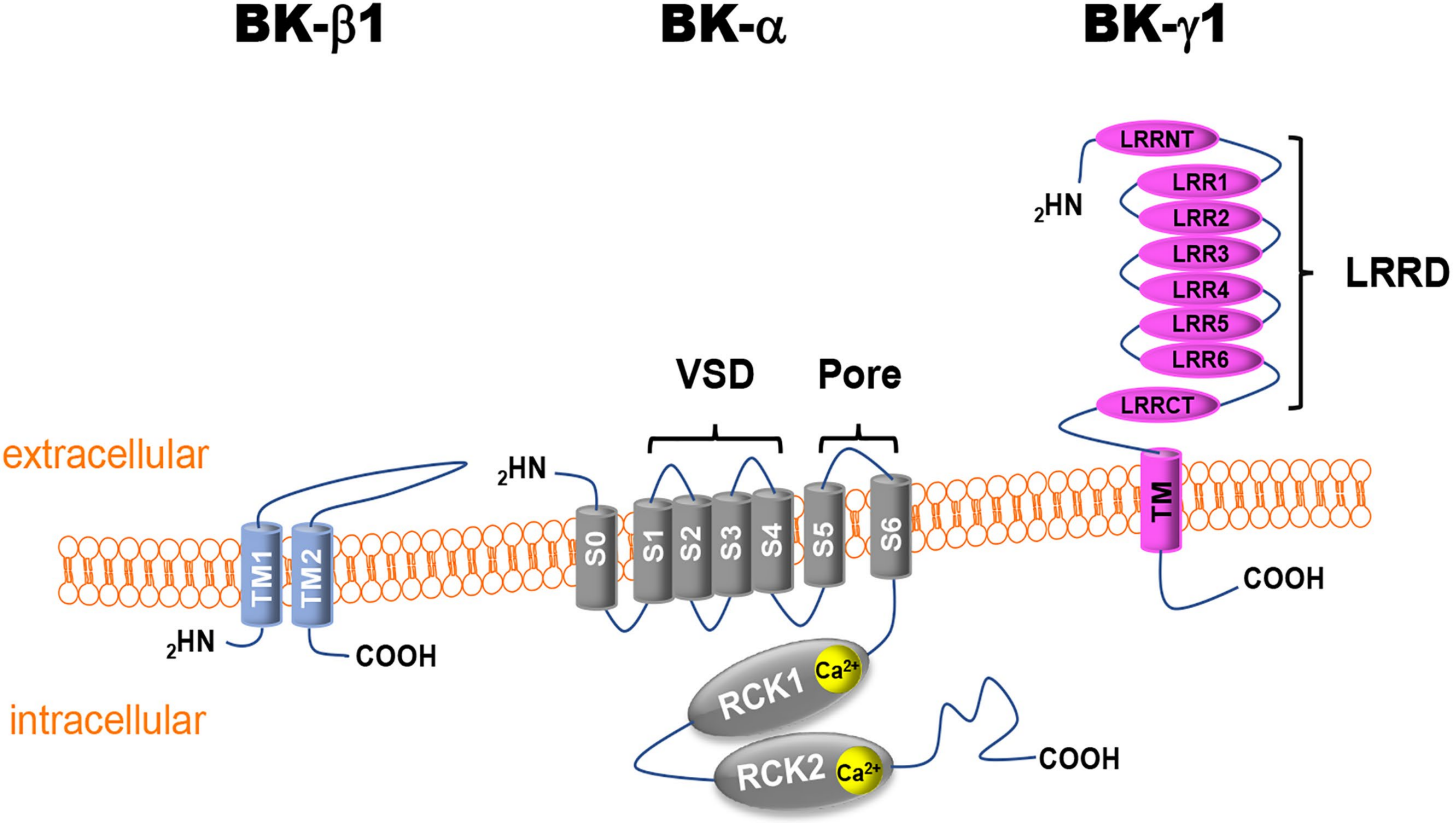
Schematic illustration of vascular Ca^2+^-activated K^+^ (BK) channel subunits. BK-α, BK channel α-subunit; BK-β1, BK channel β1-subunit; BK-γ1, BK channel γ1-subunit; S or TM, transmembrane domain segment; VDS, voltage-sensor domain; RCK, regulator of K^+^ conductance; LRR, leucine-rich repeat; LRRD, leucine-rich repeat domain; LRRCT, leucine-rich repeat C-terminus; LRRNT, leucine-rich repeat N-terminus; COOH, C-terminus; and NH_2_, N-terminus.

Cardiac perfusion is regulated by vasoactive agents released by the endothelium from mechanical sensing of luminal shear stress, including endothelium-derived relaxation factors (EDRF) and endothelium-derived hyperpolarizing factors (EDHF), the pharmacologic action of neuroendocrine factors, and the response of coronary arteriolar SMCs to intralumenal pressure (Goodwill et al., 2017). Functional vascular BK channels are composed of the pore-forming α-subunits (BK-α) and the accessory β1-subunits (BK-β1) and/or γ1-subunits (BK-γ1; Figure 1; Knaus et al., 1994; Yan and Aldrich, 2012). Four BK-α and four BK-β1 assemble to form a functional BK channel. The stoichiometry and interaction between BK-α and BK-γ1 are currently unclear. BK-α is expressed ubiquitously on the cell surface and in mitochondrial membranes of excitable and non-excitable cells, while BK-β1 is distributed in the cell membranes of excitable cells. BK-γ1 is mainly found in the cell membrane of non-excitable cells (Singh et al., 2013; Li et al., 2016). BK-α (encoded by the *KCNMA1* gene) contains the structure of six transmembrane domains (S1–S6) of voltage-gated K^+^ channels in which S1–S4 constitute the voltage-sensing domain (VSD) and the S5-P loop-S6 form the ion permeation domain, containing the conserved K^+^ selectivity filter (TVGYG; Ma et al., 2006; Cui et al., 2009). In addition, the BK channel has a unique S0 segment unit in the extracellular N-terminus and a large C-terminal domain (CTD). The CTD has four cytosolic domains (S7–S10) with two regulators of K^+^ conductance domains (RCK1 and RCK2) that contain two high-affinity Ca^2+^ binding sites (Wu and Marx, 2010; Yuan et al., 2010). One such site is the Ca^2+^ bowl (889-QFLDQDDDD-897) in RCK2 with a Ca^2+^ concentration at half-maximal effect (EC_50_) in the 10^−6^M range (Xia et al., 2002; Bao et al., 2004). The other site (D367/E535/R514) is located in RCK1 (Figure 1; Zeng et al., 2005; Zhang et al., 2010b). The RCK1s and RCK2s of four BK-α subunits form an octameric gating ring that connects to the VSD through a rigid linker (Yuan et al., 2010; Tao et al., 2017). Binding to intracellular free Ca^2+^ and membrane depolarization activate BK channels through allosteric changes in the gating ring.

In addition to Ca^2+^- and voltage-dependent activation, BK-α activity is tightly regulated by its accessory subunits, BK-β and BK-γ (Li and Yan, 2016; Gonzalez-Perez and Lingle, 2019). Four isoforms of β subunits (BK-β1-4, encoded by the *KCNMB1-4* genes) and γ subunits (BK-γ1-4, encoded by the *LRRC26*, *LRRC38*, *LRRC52*, and *LRRC55* genes) have been cloned in mammalian cells (Li and Yan, 2016; Gonzalez-Perez and Lingle, 2019). In vascular SMCs, BK-β1 is the predominant vascular isoform. It contains two transmembrane domains (TM1 and TM2) with a relatively large extracellular loop that can reach the inner mouth of the BK-α channel pore and modulates the binding of iberiotoxin (IBTX) and the effects of fatty acids on BK channel activity (Torres et al., 2014). The TM1 is thought to interact with the S2 of an adjacent BK-α subunit and the TM2 with the S0 of another adjacent BK-α subunit (Liu et al., 2010). The presence of the BK-β1 subunit enhances channel sensitivity to Ca^2+^ activation.

BK-γ1 is also expressed in vascular SMCs (Evanson et al., 2014). BK-γ1 shares the structure of the leucine-rich repeat (LRR) protein superfamily and contains an extracellular N-terminus with six LRRs, a single transmembrane domain, and a short intracellular C-terminus (Figure 1). The effects of BK-γ1 on BK-α regulation can be reproduced by a 40-amino acid peptide containing the transmembrane domain of BK-γ1, suggesting that this is an important structure in the regulation of BK channel physiology (Li et al., 2016). BK-γ1 is known to enhance BK-α sensitivity to Ca^2+^ and voltage stimuli by magnitudes similar to those of BK-β1, allowing BK channel activation in the physiological range of intracellular free Ca^2+^ concentrations and membrane potentials of vascular SMCs (Tanaka et al., 1997; Cox and Aldrich, 2000; Yan and Aldrich, 2012). In heterologous expression systems, BK-β and BK-γ subunits can co-exist in the same functional BK channel complex. Their effects on the intrinsic properties of the channel were additive, suggesting that the multiplicity of BK-β/BK-γ combinations would generate a range of BK channels with distinct functional properties according to the specific stoichiometry of the contributing subunits (Gonzalez-Perez et al., 2015). Since nothing is known about the role of BK-γ in the regulation of coronary BK channels in DM, this review will focus on the findings regarding BK-α and BK-β1 pathophysiology in DM.

Intracellular Ca^2+^ homeostasis in vascular SMCs is regulated by the balance between sarcolemmal Ca^2+^ entry (L-type Ca^2+^ channels and the transient receptor potential channels; TRP, etc.), release of Ca^2+^ from the endoplasmic reticulum/sarcoplasmic reticulum, uptake of cytoplasmic Ca^2+^ into intracellular stores, and extrusion through the sarcolemmal Ca^2+^ pump and Na^+^/Ca^2+^ exchanger (Leopold, 2015). In vascular SMCs, BK channels link Ca^2+^ homeostasis with cellular excitability and regulate vascular tone through membrane hyperpolarization, providing a negative feedback mechanism on Ca^2+^ entry. BK channels are colocalized with L-type Ca^2+^ channels and TRPC/TRPV channels to form BK channel-Ca^2+^ signaling complexes in the sarcolemma of vascular SMCs, allowing channel regulation in the local cellular milieu (Earley et al., 2005; Kwan et al., 2009; Suzuki et al., 2013; Hashad et al., 2018). Activation of L-type Ca^2+^ channels and TRP channels in vascular SMCs produces Ca^2+^ sparklets and triggers Ca^2+^ release from the SR to generate Ca^2+^ sparks (Nelson and Quayle, 1995; Takeda et al., 2011). With a single channel conductance of ~300pS, BK channels contribute to 50% of the total K^+^ currents in coronary arterial SMCs (Wang et al., 2011; Sun et al., 2020). Activation of vascular BK channels by Ca^2+^ sparks/sparklets in their vicinity gives rise to spontaneous transient outward currents (STOCs), which hyperpolarize the cellular membrane potentials, inactivate L-type Ca^2+^ channels and TRP channels, reduce intracellular Ca^2+^ concentrations, and lead to vasorelaxation (Nelson et al., 1995; Ledoux et al., 2006). In addition, BK channels are also expressed in vascular endothelial cells (ECs). Activation of endothelial BK channels may hyperpolarize adjacent SMCs, bestowing EDHF effects (Bryan et al., 2005; Hughes et al., 2010). Nevertheless, activation of BK channels contributes to more than 70% of total vasodilation induced by bradykinin (Miura et al., 1999) and 40% of total vasodilation induced by shear stress in human coronary resistance vessels (Lu et al., 2019).

## Coronary BK Channel Dysfunction in DM

Both T1DM and T2DM are known to be independent risk factors for cardiovascular diseases, and cardiovascular diseases continue to be a leading cause of mortality in diabetic patients (Dhalla et al., 1985; Stone et al., 1989; Brindisi et al., 2010; Leon and Maddox, 2015). Although, the prevalence of cardiovascular disease in the general population has decreased by 35–40% over recent decades, such a decline has not been observed in patients with DM (Gregg et al., 2007; Beckman and Creager, 2016; Cefalu et al., 2018). Endothelial dysfunction has been recognized as the mechanism that underlies vascular pathology of DM. Subsequent findings confirm that vascular smooth muscle dysfunction is equally important in the pathophysiology of diabetic cardiovascular complications (Creager et al., 2003).

**Figure 2:**
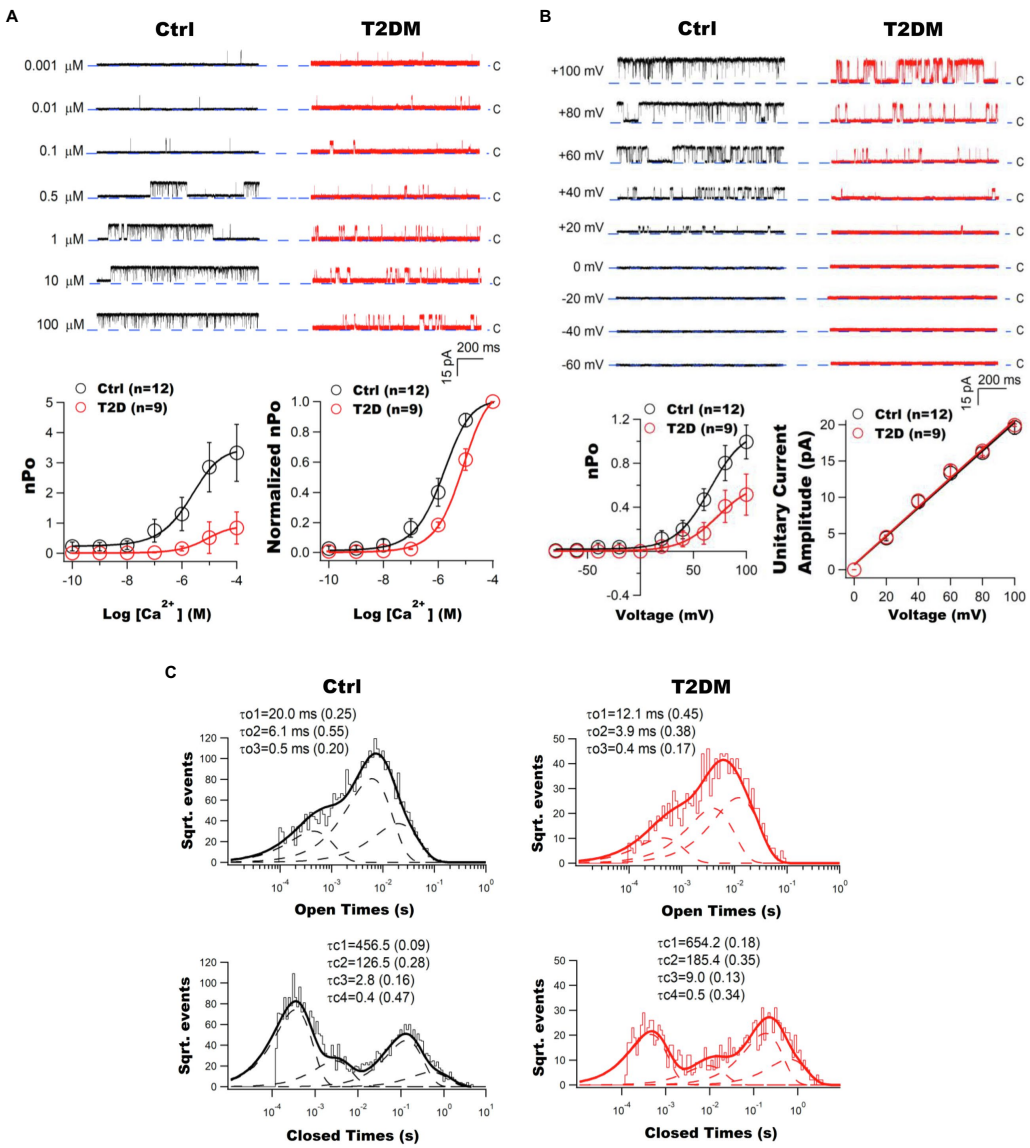
Impaired vascular BK channel function in patients with T2DM. **(A)** Coronary arterioles of T2DM patients exhibit diminished BK channel Ca^2+^ sensitivity. Left panel: Representative tracings of inside-out single BK channel currents recorded at +60mV in an excised patch of freshly isolated atrial coronary arteriolar myocytes from non-diabetic (Ctrl) and T2DM patients. With an increase in free Ca^2+^ concentration, BK channel open probability (nPo) was robust in controls but not in T2DM patients. Dashed lines indicate the closed state (c) of channel. Right panel: The nPo plotted against logarithm Ca^2+^ concentrations (nPo-log[Ca^2+^] curve) was fitted using the Hill equation. There were significant reductions in Ca^2+^ log[EC_50_] and BK channel maximal nPo in T2DM patients (*n*=9) compared to those in non-diabetic controls (*n*=12). A rightward shift on the normalized nPo-log[Ca^2+^] curve of T2DM patients. Data are presented as mean±SEM. The BK channel maximal nPo and log[EC_50_] were significantly reduced in diabetic patients. **(B)** Impaired BK channel voltage sensitivity in the coronary arterioles of T2DM patients. Left panel: Representative tracings of inside-out single BK channel currents elicited at different testing voltages in the presence of 200nM free Ca^2+^ in freshly isolated coronary arteriolar smooth muscle cells (SMCs) from non-diabetic controls and T2DM patients. BK channel was activated by membrane depolarization with reduced effects in diabetes mellitus (DM). The dashed line indicates the closed state (c) of channel. Right panel: BK channel open probability and voltage (nPo–V) relationships were fitted using the Boltzmann equation. The maximal nPo and voltage at half of maximal channel activation (V_0.5_) were significantly decreased in T2DM patients (*n*=9), compared with controls (*n*=12). BK channel unitary current amplitude plotted against membrane voltages (i–V curves) were fitted using a linear equation. The unitary conductance of BK channels was not different between controls and T2DM patients. Data are presented as mean±SEM. There was a significant decrease in BK channel maximal nPo and V_0.5_ in diabetic patients. **(C)** Altered BK channel kinetics in the coronary arterioles of T2DM patients. Typical histograms of BK channel open and closed dwell-time durations are illustrated. Data were obtained from inside-out patches at +60mV in the presence of 200nM free Ca^2+^ in the bath solution. Dwell-time distributions were best fitted by the sum of exponential probability density functions with three open time constant components (the slow τo1, the intermediate τo2, and the fast τo3) and four closed time constant components (the very slow τc1, the slow τc2, the intermediate τc3, and the fast τc4). Dashed lines represent the distribution of exponential components determined by the logarithm likelihood ratio test. The values of each time constant component and its relative weight (in parentheses) are given above each histogram. This figure was adapted from published results with the permission of Cardiovascular Research (Lu et al., 2019).

Impaired BK channel-induced vasodilation was first discovered in the cerebral arteries of fructose-rich diet-induced insulin-resistant rats (Dimitropoulou et al., 2002; Erdos et al., 2002). Patch clamp studies provided direct evidence of BK channel dysfunction in freshly isolated coronary arterial SMCs from Zucker diabetic fatty (ZDF) rats, a genetic animal model of T2DM (Lu et al., 2005). Abnormal vascular BK channel function was also found in other diabetic animal models, including streptozotocin (STZ)-induced T1DM rodents, db/db T2DM mice, high fat diet (HFD)-induced obesity/diabetic mice and swine (Dimitropoulou et al., 2002; Pietryga et al., 2005; Burnham et al., 2006; McGahon et al., 2007; Yang et al., 2007; Dong et al., 2008; Lu et al., 2008, 2010, 2012, 2016, 2017a; Borbouse et al., 2009; Navedo et al., 2010; Zhang et al., 2010a; Mori et al., 2011; Nystoriak et al., 2014; Yi et al., 2014). It is worth noting that diabetic vascular BK channel dysfunction is a common finding in most vascular beds, but the results can vary in different species, animal models, and disease status (Mokelke et al., 2003, 2005; Christ et al., 2004; Pietryga et al., 2005; Burnham et al., 2006; Davies et al., 2007; McGahon et al., 2007; Lu et al., 2008; Borbouse et al., 2009; Navedo et al., 2010; Mori et al., 2011; Rueda et al., 2013; Nystoriak et al., 2014; Nieves-Cintron et al., 2017). It has been found that in freshly isolated coronary arterioles from patients with T2DM, BK channel sensitivity to Ca^2+^ and voltage activation was reduced, indicating that the intrinsic biophysical properties of BK channels were altered in diabetic patients (Figure 2; Lu et al., 2019). This finding supports the observation that the BK channel response to Ca^2+^ sparks was diminished in human diabetic vessels. The significance of coronary BK channel dysfunction in DM is underscored by the finding that ischemia–reperfusion-mediated myocardial infarction is exacerbated in STZ-induced T1DM mouse hearts and can be reproduced in non-diabetics hearts after exposure to the BK channel specific inhibitor, iberiotoxin (IBTX; Lu et al., 2016). Since IBTX is membrane impermeable and cardiac myocytes do not have BK-a expression on the sarcolemma, this finding provides evidence of the role of coronary vascular BK channels on cardioprotection during ischemia–reperfusion insults, as well as the loss of its protection in DM.

### Altered BK Channel Protein Expression in Diabetic Vessels

Altered coronary vascular BK channel expression is common in DM (Burnham et al., 2006; McGahon et al., 2007). However, diverse levels of vascular BK channel expression in DM have been observed. In most case, the protein expressions of BK channels are downregulated in coronary arteries (Burnham et al., 2006; Dong et al., 2008; Lu et al., 2008, 2017a; Zhang et al., 2010a; Rueda et al., 2013; Nystoriak et al., 2014; Li et al., 2017), but it was reportedly increased, despite impaired BK channel function in the coronary arteries of Ossabaw miniature swine with metabolic syndrome (Borbouse et al., 2009). Recently, human BK channel expression was examined in coronary arterioles obtained from atrial biopsies of patients who underwent coronary artery bypass grafting surgery. Protein downregulation was found in both BK-α and BK-β1 in patients with T2DM, compared to age-matched non-diabetic subjects (Lu et al., 2019). However, the mRNA levels of BK-β1 were (McGahon et al., 2007) not reduced in the coronary arteries of STZ-induced T1DM rats (Zhang et al., 2010a), db/db T2DM mice (Li et al., 2017) and HFD-induced diabetic mice (Lu et al., 2017a). The varied reports of BK channel expression suggest that a complex assortment of mechanisms exist in the regulation of vascular BK channel expression and function in DM. Reduced BK channel expression leads to impaired Ca^2+^ sparks/STOCs coupling, albeit the Ca^2+^ spark amplitudes and intracellular Ca^2+^ concentrations are known to be elevated in diabetic vascular SMCs.

### Impaired BK Channel Biophysical Properties and Kinetics in Coronary Arterial SMCs in DM

Ca^2+^-activated K^+^ channel currents (I) are determined by the number of activated channels (N), open probability (Po), and channel unitary conductance (*i*), where I=N^*^Po^*^i. BK channel current density is reduced in the coronary arteries of T1DM and T2DM animal models and in humans with DM (Lu et al., 2005, 2008, 2010, 2012, 2016, 2017a, 2019; Pietryga et al., 2005; Burnham et al., 2006; McGahon et al., 2007; Dong et al., 2008; Zhang et al., 2010a; Nystoriak et al., 2014; Yi et al., 2014; Li et al., 2017; Nieves-Cintron et al., 2017; Tang et al., 2017; Zhang et al., 2020). BK channels are activated by intracellular free Ca^2+^ concentration and by membrane depolarization (Cox et al., 1997; Lu et al., 2008), and these are impaired in DM (Lu et al., 2008, 2019). BK channel sensitivity to voltage- and Ca^2+^-mediated activation can be measured by using inside-out patch clamp studies in which the excised cell membrane can be clamped to various voltages and the cytoplasmic surface of the cell membrane directly exposed to bath solutions containing various free Ca^2+^ concentrations. In freshly isolated coronary arterial SMCs of ZDF rats at 8months after the development of hyperglycemia, BK channels had a rightward-shifted Ca^2+^ concentration-dependent curve, with increased EC_50_ for Ca^2+^ activation and decreased Ca^2+^ cooperativity, compared to those of Lean control rats (Lu et al., 2008). Moreover, BK channel activation by membrane depolarization was also abnormal in coronary arterial SMCs of ZDF rats. The channel open probability–voltage (Po-V) relationships were rightward and downward shifted, with the voltage at 50% maximal Po increased by 40mV. These results indicate that a higher cytoplasmic Ca^2+^ concentration and a more depolarized membrane potential are required to activate BK channels in DM. Changes in the intrinsic free energy of Ca^2+^-binding (ΔΔCa^2+^) that contributes to BK channel activation can be estimated based on the shift of Po-V relationship from 0 to 1 μM free Ca^2+^ in Lean and ZDF rats using the equation: ΔΔCa^2+^=−Δ(*ze*V_0.5_), where *z* is the number of equivalence charge movement, *e* is the elementary charge, and V_0.5_ is the voltage at half maximal activation (Shi et al., 2002). There was a 62.3% decrease in the ΔΔCa^2+^ in ZDF rats, suggesting a less favorable condition for Ca^2+^ binding to vascular BK channel Ca^2+^ sensors in ZDF rats (Lu et al., 2008). Similar results were also observed in BK channels in freshly isolated coronary microvascular SMCs from the atrial appendages of patients with T2DM. Ca^2+^- and voltage sensitivity were significantly impaired in diabetic patients, with the maximal BK channel activity to free Ca^2+^ and voltage activation reduced by 70 and 50%, respectively (Figure 2; Lu et al., 2019). Such dysregulation contributed to a 27.4% attenuation in shear stress-mediated coronary arteriolar vasorelaxation in diabetic patients compared with non-diabetic controls (Lu et al., 2019). In addition, single BK channel current amplitudes were unaltered in DM, indicating that the conductance property of vascular BK channels is normal in DM.

Vascular large conductance Ca^2+^-activated K^+^ channel gating kinetics contain multiple components of open and closed states and dwell-times (McManus and Magleby, 1988, 1991). In coronary arterial SMCs, the open and closed dwell-time histograms of single BK channels were best fitted with three open-time constants: fast (τo_1_), intermediate (τo_2_), and slow (τo_3_), along with four closed-time constants: fast (τc_1_), intermediate (τc_2_), slow (τc_3_), and very slow (τc_4_). DM affects both channel open dwell-times and channel closed dwell-times. The BK channel mean closed-time constant and the individual closed-time constants were significantly prolonged. At the same time, the channel mean open-time constant and individual open-time constants were significantly reduced in DM. These findings were seen in both ZDF rats and in diabetic patients (Lu et al., 2008, 2019). These changes in BK channel gating kinetics suggest that channel openings are abbreviated, and closures prolonged in DM, with reduced channel Po and maximal activation. Hence, diabetes not only affects BK channel expression, but also alters the intrinsic biophysical properties of the channel.

### *KCBMA1* and *KCNMB1* Variations Associated With Obesity and DM

Genome-wide association studies (GWASs) are a powerful tool to find genetic variations associated with diseases. Results from a few studies have shown a strong association between *KCNMA1* splicing variants and the incidence of obesity or DM. The results from case–control cohorts involving 4,838 obese and 5,827 control subjects suggested that the *KCNMA1* rs2116830*G variant was associated with obesity with a *p* value of 2.82×10^−10^ (Jiao et al., 2011). A recent study reported that a *de novo* missense variant in *KCNMA1* (c.1123G>A) was identified in an adult male patient with a plethora of developmental phenotypes including neonatal DM. This loss-of-function polymorphism (p. G375A) of BK channel is located in the S6 transmembrane domain of BK channel (Liang et al., 2019). In addition, it is well known that BK-α and BK-β1 undergo extensive alternative pre-mRNA splicing and that these splice variants have significant changes in BK channel intrinsic properties and surface expression (Poulsen et al., 2009). However, the pathophysiological roles of BK channel variants in the development of BK channelopathy in DM are largely unexplored and warrant further investigation.

## Signaling Molecules and Pathways Mediating Vascular BK Channel Dysfunction in DM

### Effects of Reactive Oxygen Species on Vascular BK Channel Redox Modification

Increased reactive oxygen species (ROS) production is a hallmark of diabetic pathophysiology, and the role of ROS on vascular dysfunction has been extensively reviewed (Inoguchi et al., 2003; Konior et al., 2014). ROS is represented by a group of highly reactive molecules that include superoxide anion (O_2_^•–^), peroxide ion (O_2_^2−^), hydrogen peroxide (H_2_O_2_), and peroxynitrite (ONOO^−^). In vascular SMCs, multiple enzymatic systems such as the NADPH oxidases (NOXs), xanthine oxidase (XO), nitric oxide synthases (NOS), and the mitochondrial electron transport chain are known to produce O_2_^•–^ and H_2_O_2_ (Taniyama et al., 2004; Byon et al., 2016). The NOXs, in particular NOX1 and NOX4, are the most important because they are commonly expressed in vascular cells and are the major source of ROS generation in vessels (Clempus and Griendling, 2006; Konior et al., 2014; Burtenshaw et al., 2017). O_2_^•–^ is converted to H_2_O_2_ by superoxide dismutases (SODs) or reacts with nitric oxide (NO) to form ONOO^−^. H_2_O_2_ is further reduced to H_2_O by catalase (CAT) and glutathione peroxidase (GPx; Taniyama and Griendling, 2003). Oxidative stress due to ROS production outweighing their scavenging is implicated in vascular dysfunction associated with T1DM and T2DM. It is well documented that elevated glucose increases the production of intracellular advanced glycation end-products (AGEs), stimulates the protein kinase C (PKC)-dependent activation of NOX1 and NOX4 (Inoguchi et al., 2000; Lu et al., 2006; Deluyker et al., 2017), and reduces the activity and bioavailability of antioxidant enzymes, such as SODs, GSH, CAT, and GPx, which results in higher ROS levels in both vascular ECs and SMCs in DM (Szaleczky et al., 1999; Lu et al., 2012; Tiwari et al., 2013).

Reactive oxygen species triggers many signaling pathways and promotes redox-mediated protein posttranslational modification. We found that redox modification is involved in BK channel dysfunction through hyperglycemia. High glucose culture of HEK293 cells stably expressing BK-α resulted in altered BK-α activity and channel kinetics that were mimicked by the effects of exogenously applied H_2_O_2_ in BK-α expressing cells cultured in normal glucose (Lu et al., 2006). A 1-week culture with 22mM glucose markedly downregulated the protein expression of CAT and CuZn-SOD in HEK293 cells, leading to a 3.3-fold increase of H_2_O_2_ concentration to the 10^−3^M range. Consequently, high glucose culture produced a 50% reduction of BK-α current density, prolonged the channel activation and deactivation time constants (τ_A_ and τ_D_), and upward shifted the τ-V curve, indicating that BK-α activation is suppressed in high glucose conditions (Lu et al., 2006). The effects of high glucose on BK-α voltage-dependent activation were mimicked by acute exposure to 2mM H_2_O_2_. Furthermore, the cysteine residue at 911 (C911) in BK-α is particularly vulnerable to H_2_O_2_-mediated regulation (Tang et al., 2001), and a single substitution of C911 by alanine (C911A) eliminated most of the inhibitory effects of BK-α under high glucose conditions and to exogenously applied H_2_O_2_ (Lu et al., 2006). In addition, acute exposure to ONOO^−^ (5–100μM) significantly suppressed BK channel activity in vascular SMCs (Brzezinska et al., 2000; Liu et al., 2002), but did not alter BK-α voltage-dependent activation (Lu et al., 2006), suggesting that the molecular mechanisms underlying BK channel regulation by H_2_O_2_ and ONOO^−^ are different. Further studies revealed a 3- to 4-fold increase of 3-nitrotyrosine levels on BK-α protein in freshly isolated aortas from STZ-induced T1DM rats compared to non-diabetic controls, suggesting that ONOO^−^-induced modification of BK-α may be mediated through protein tyrosine nitration rather than protein oxidation (Lu et al., 2010). The precise amino acid residue(s) in BK-α modified by ONOO^−^ has not been identified. Nevertheless, an increase of ROS accumulation is the culprit for the development of BK channel dysfunction in DM.

### Angiotensin II Signaling and Vascular BK Channel Regulation

**Figure 3:**
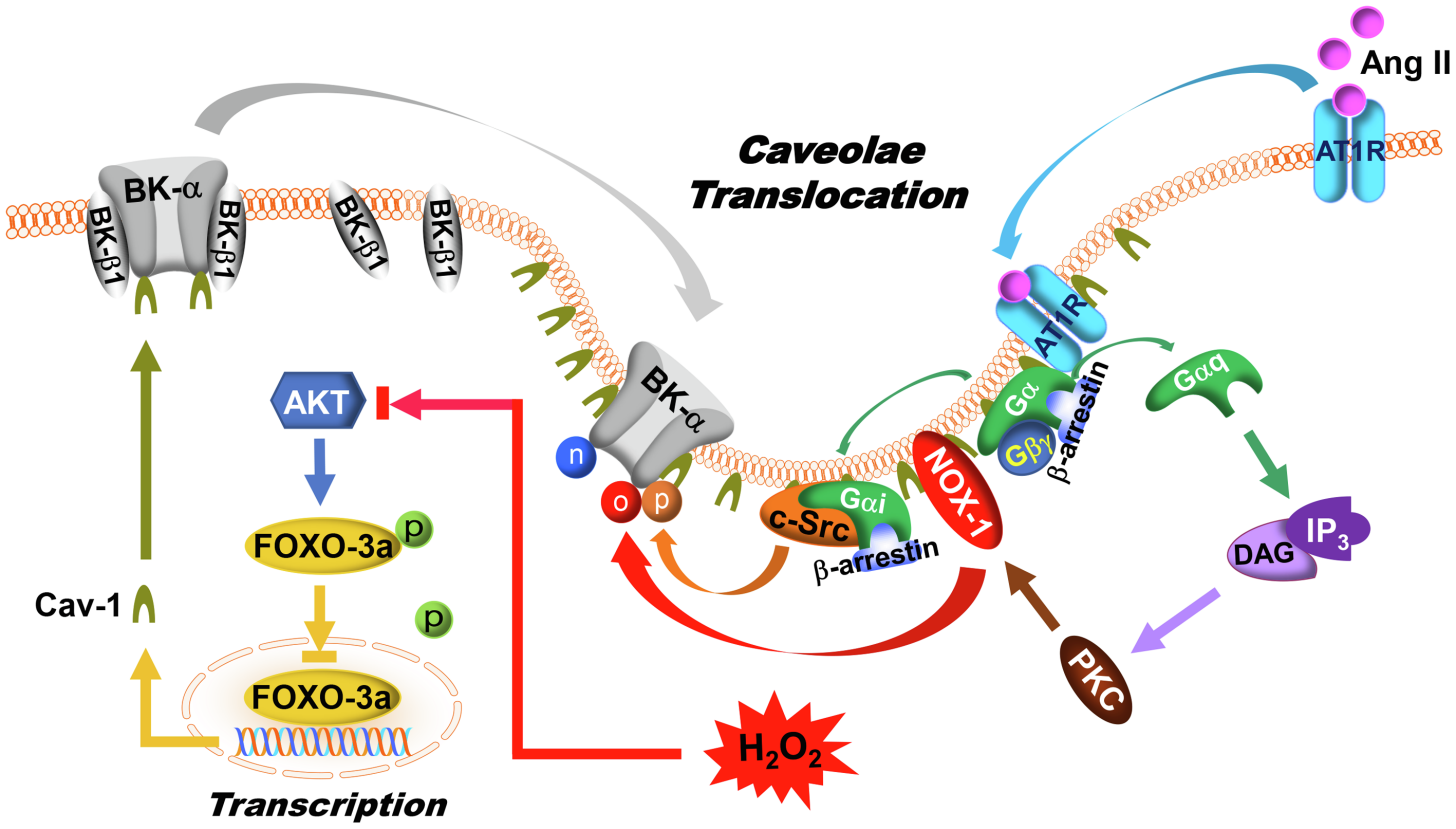
Regulation of BK channels by AT1R signaling and caveolae compartmentalization. In DM, AT1R expression, and caveolae formation are upregulated in vascular SMCs. Upon Ang II activation, AT1R translocates to caveolae, where G-proteins, BK-α, NOX-1, and c-Src are colocalized. In caveolae, AT1R interacts with Gαq to activate PKC and NOX-1 through IP_3_/DAG signaling pathway, leading to an increase of ROS production. Meanwhile, the Gαi and β-arrestin complex induces c-Src activation. As a result of AT1R activation, BK-α protein oxidation, tyrosine phosphorylation, and tyrosine nitration are enhanced. In addition, AKT phosphorylates FOXO-3a, which in turn suppresses FOXO-3a nuclear translocation and reduces its transcriptional activities. With high glucose, increased ROS production inhibits AKT function, which promotes FOXO-3a nuclear translocation and facilitates Cav-1 expression. Since BK-β1 is not present in the caveolae, an increase in BK-α compartmentalization in caveolae may lead to physical uncoupling between BK-α and BK-β1 in vascular SMCs. The symbols “n,” “o,” and “p” represent protein nitration, oxidation, and phosphorylation, respectively.

Angiotensin II (Ang II) is an oligopeptide hormone, exerting its physiological and pathophysiological effects through binding to Ang II type 1 (AT1R) and type 2 (AT2R) receptors and activating their downstream signaling pathways (Dasgupta and Zhang, 2011). In vascular SMCs, where AT1R is predominantly expressed, Ang II causes vasoconstriction and promotes vascular wall remodeling (Ribeiro-Oliveira et al., 2008). In contrast, activation of AT2R produces vasodilatation and impairs vascular remodeling, effects opposite to those of AT1R (Danyel et al., 2013). AT1R is a G-protein-coupled receptor, which is coupled to Gαq, Gβγ, Gαi, and β-arrestin (Kawai et al., 2017; Wang et al., 2018). Binding of Ang II to AT1R in vascular SMCs activates Gαq which in turn activates the phospholipase C (PLC)-dependent inositol-1,4,5-triphosphate (IP_3_)/diacylglycerol (DAG)-mediated Ca^2+^ signaling cascades, causing an increase in protein kinase C (PKC) activity (De Gasparo et al., 2000; Touyz and Schiffrin, 2000). Activation of PKCβ stimulates NOXs with ROS overproduction under hyperglycemic conditions (Inoguchi et al., 2000; Evcimen and King, 2007) and is a cause of impaired vascular BK channel function in diabetic vessels (Figure 3; Zhou et al., 2006; Lu et al., 2012; Zhang et al., 2020). In addition to redox-mediated modification of BK-α, it has been shown that PKC-induced serine phosphorylation at 695 (S695) and 1151 (S1151) in the C-terminus of BK-α inhibits BK channel current density by 50%, and S1151 phosphorylation by PKC also abolishes BK-α activation by protein kinase A (PKA) and protein kinase G (PKG; Zhou et al., 2001, 2010). On the other hand, the activity of tyrosine-protein kinase is regulated by Gαi and β-arrestin upon AT1R stimulation, causing BK channel dysfunction (Ma et al., 2000; Alioua et al., 2002; Fessart et al., 2005; Tian et al., 2007). Another study reported that the C-terminus of AT1R physically interacts with the C-terminus of BK-α in heterologous expression system, and such protein–protein interaction between AT1R and BK-α directly inhibits BK-α activity, independent of G-protein mediated processes (Zhang et al., 2014).

However, AT1R expression, Ang II bioavailability, and tissue sensitivity to Ang II are upregulated in diabetic vessels (Arun et al., 2004; Kawai et al., 2017). The pathophysiological importance of Ang II-mediated BK channel regulation in diabetic coronary arteries is supported by the evidence that cardiac infarct size induced by experimental ischemia/reperfusion in STZ-induced T1DM mice was twice as large as non-diabetic mice (Lu et al., 2016). The effects of DM on myocardial ischemia/reperfusion injury can be reproduced by infusion of 2μM Ang II or 0.1μM membrane impermeable BK channel inhibitor, IBTX, but attenuated by the BK channel activator, NS-1619 (Lu et al., 2016). Similar results were observed in Akita T1DM mice with exacerbated cardiovascular complications and cardiac and vascular dysfunction, from an imbalance of Ang II/AT1R signaling in DM (Patel et al., 2012). Most importantly, the pathological roles of Ang II signaling are supported by clinical outcomes showing that treatment with AT1R blockers and ACE inhibitors reduced cardiovascular complications and cardiovascular death in patients with DM by 25–30% (Niklason et al., 2004; Abuissa et al., 2005; Cheng et al., 2014; Lv et al., 2018).

### Caveolae Compartmentation and Vascular BK Channel Subcellular Distribution

Caveolae, which are nonclathrin-coated, flask-shaped invaginations of plasma membrane lipid raft subdomains, are characterized by their signature structural protein caveolin, with caveolin-1 (Cav-1) predominantly expressed in the vasculature (Gratton et al., 2004; Krajewska and Maslowska, 2004). Caveolae have emerged as a central platform for signal transduction in many tissues through the interaction between the Cav scaffolding domain and protein partners that contain a Cav-binding motif (ΦxΦxxxxΦ or ΦxxxxΦxxΦ, where Φ is an aromatic amino acid, and x is any amino acid; Okamoto et al., 1998). Many signaling molecules that are associated with BK channel regulation, such as the β-adrenergic receptors (Bucci et al., 2004), AT1R (Ushio-Fukai and Alexander, 2006; Basset et al., 2009), NOX1 (Hilenski et al., 2004; Wolin, 2004), cellular tyrosin protein kinase Src (c-Src; Zundel et al., 2000; Lee et al., 2001), guanylyl cyclase (Linder et al., 2005; Vellecco et al., 2016), PKA (Heijnen et al., 2004; Linder et al., 2005), protein kinase B (PKB or AKT; Sedding et al., 2005), PKC (Zeydanli et al., 2011; Ringvold and Khalil, 2017), PKG (Linder et al., 2005), NOS (Garcia-Cardena et al., 1996; Vellecco et al., 2016), and prostacyclin (PGI_2_) synthase (PGIS; Spisni et al., 2001), are found in the low buoyant density, caveolae-rich membrane fractions of vascular ECs and SMCs. The significance of Cav-1 on vascular physiology is demonstrated by findings in Cav-1 knockout (KO) mice that show constitutively activated eNOS with elevated NO production as well as a failure to maintain a constant vasocontractile tone, resulting in the development of cardiovascular pathologies (Drab et al., 2001; Razani et al., 2001). Overgeneration of NO facilitates the production of ONOO^−^ and contributes to vascular dysfunction with excessive H_2_O_2_ accumulation (Pacher et al., 2007).

The consensus sequence of the Cav-binding motif is present in BK-α, but not in BK-β1. Indeed, only BK-α but not BK-β1 is detected in the caveolae-rich fractions of SMCs (Lu et al., 2016). Moreover, BK-α is colocalized in the caveolae with other ion channels (Wang et al., 2005; Riddle et al., 2011; Howitt et al., 2012; Lu et al., 2016), especially those associated with Ca^2+^ spark/sparklet generation, such as L-type Ca^2+^ channels (Suzuki et al., 2013; Saeki et al., 2019), T-type Ca^2+^ channels (Hashad et al., 2018), TRPV4 (Goedicke-Fritz et al., 2015; Lu et al., 2017b), TRPC1, TRPC3, and TRPC6 (Bergdahl et al., 2003; Adebiyi et al., 2011; Grayson et al., 2017) in vascular ECs and SMCs. The close proximity of BK channels with Ca^2+^ entry molecules leads to Ca^2+^ spark-coupled STOCs. However, it has been reported that Cav-1 interacts with BK channels and inhibits BK channel activities in coronary ECs (Wang et al., 2005; Riddle et al., 2011). Cholesterol depletion by methyl-β-cyclodextrin and silencing of Cav-1 by small interference RNA enhance BK currents, while exposure to the scaffolding domain peptide of Cav-1 (AP-CAV) inhibits BK currents (Wang et al., 2005; Riddle et al., 2011). Hence, the presence of caveolae may exert an inhibitory effect on BK channel activity.

Increased Cav-1 expression has been found in most diabetic vessels (Hillman et al., 2001; Bucci et al., 2004; Pascariu et al., 2004; Elcioglu et al., 2010; Uyy et al., 2010; Li et al., 2014). Cav-1 expression is directly upregulated by the Forkhead Box O (FOXO) transcription factor (Sandri et al., 2004; Van Den Heuvel et al., 2005). The FOXO-3a phosphorylation levels are significantly reduced in STZ-induced T1DM rat arteries and in cultured human coronary arterial SMCs (Zhang et al., 2010a). This explains the underlying mechanism that leads to Cav-1 upregulation in DM (Figure 3). Furthermore, in STZ-induced T1DM rats, our results in co-immunoprecipitation experiments show that AT1R, c-Src, and BK-α are enriched in the low buoyant density, caveolae-rich membrane fractions of aortas, compared to non-diabetic rats (Lu et al., 2010). Infusion with Ang II (0.05μg/kg) results in markedly enhanced AT1R protein translocation to the low buoyant density fractions of aortas after 1h (83.4% of total membrane AT1R in STZ-induced T1DM rats vs. 28.5% in controls), suggesting enhanced AT1R translocation into caveolae-rich lipid rafts upon agonist activation in diabetic vessels, consistent with previous report in cultured vascular SMCs (Ishizaka et al., 1998). However, the precise mechanism underlying AT1R translocation is currently unclear. The levels of vascular BK-α protein oxidation, tyrosine phosphorylation, and tyrosine nitration are significantly increased in STZ-induced T1DM rats, likely due to the co-localization of NOS, NOX1 and c-Src in the caveolae. Since BK-α but not BK-β1 is present in caveolae, BK-α translocation into the caveolae of arteries in STZ-induced T1DM mice may promote the physical dissociation of BK-α and BK-β1 (Lu et al., 2016), which may explain the uncoupling of BK-α and BK-β1 in diabetic vessels. A working framework has emerged in caveolae targeting of BK channel regulation, in which caveolae compartmentalize BK-α with AT1R, NOS, NOXs, and c-Src to form BK-α-receptor-enzyme microdomain complexes in vascular SMCs (Figure 3). Such caveolae compartmentation is enhanced in diabetic vessels, which facilitates the redox modification of BK-α. Of note, because BK-β1 does not translocate into caveolae, such subcellular distribution of BK-α and BK-β1 may contribute to BK-α and BK-β1 functional uncoupling, thereby exacerbating BK channelopathy in diabetic vessels (Figure 3). Additionally, caveolae take part in endosomal trafficking and regulating surface expression of many membrane proteins (Elkin et al., 2016). Taking into account the consequences of upregulation of caveolae formation in the vascular SMCs in DM, BK-α caveolae translocation may have important pathophysiological implications for vascular BK channel dysfunction in DM.

### Ubiquitin Proteasome System and Vascular BK Channel Protein Degradation

**Figure 4:**
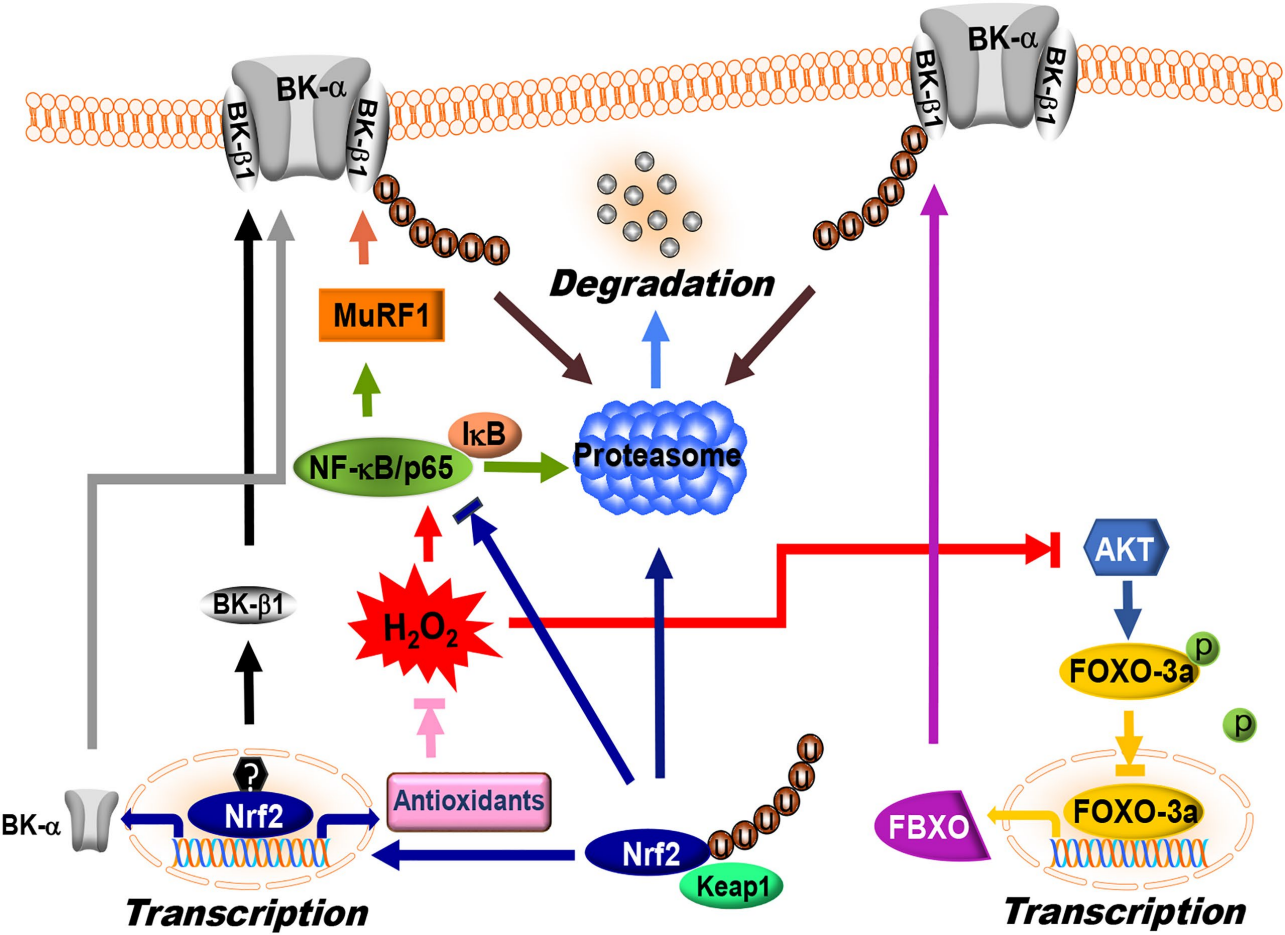
Regulation of BK channel expression by ubiquitin proteasome system (UPS) and nuclear factor erythroid-2-related factor 2 (Nrf2) signaling. FBXO and MuRF1 are the E3 ligases targeting BK-β1 protein degradation *via* the UPS in vascular SMCs. FBXO is one of downstream targets of FOXO-3a. FOXO-3a activity is negatively controlled by AKT-dependent phosphorylation, while MuRF1 expression is controlled by NF-κB/p65. Under baseline conditions, p65 is bound to an inhibitory subunit, IκB that keeps it sequestered in an inactive state in the cytoplasm. Phosphorylation of IκB by IκB kinase promotes IκB degradation through the UPS, which in turn releases p65 and facilitates nuclear translocation. Under hyperglycemic conditions, overproduction of ROS inhibits AKT and activates NF-κB/p65, which in turn promotes FBXO and MuRF1 expression, leading to BK-β1 ubiquitination and accelerated degradation in vascular SMCs. Nrf2 is the master regulator of the antioxidant response. Under normal conditions, each molecule of Nrf2 interacts with two molecules of Keap1 resulting in UPS-mediated degradation. ROS modifies specific cysteine residues in Keap1 and releases Nrf2 from binding with Keap1. The unbound Nrf2 translocates into the nucleus and binds to the promoter region of target genes. Nrf2 directly upregulates BK-α mRNA expression *via* binding to the promoter region of *KCNMA1*. However, BK-β1 mRNA expression is not regulated by Nrf2 but by other transcription factor(s). In DM, Nrf2 expression and function is significantly downregulated, leading to a decrease in BK-α expression through reduced transcription and a decrease in BK-β1 expression through accelerated UPS degradation. The symbols “u” and “p” represent protein ubiquitination and phosphorylation, respectively.

Protein homeostasis with a balanced regulation between synthesis and degradation is essential for the maintenance of normal cellular function. Cellular proteins are degraded mainly through the lysosomes and the ubiquitin proteasome system (UPS; Ciechanover, 2005). Lysosomal protein degradation occurs through fusion with endocytotic vesicles. This mechanism of protein degradation is non-specific, and all proteins are digested indiscriminately at the same rate. UPS-mediated protein degradation accounts for 80–90% of protein degradation in mammalian cells and it is substrate-specific (Powell, 2006; Schapira et al., 2019). This process is facilitated by three distinct enzymatic steps that involve an ubiquitin-activating enzyme (E1), a ubiquitin-conjugating enzyme (E2), and a ubiquitin ligase (E3). E1 interacts with ubiquitin through an E1-ubiquitin thioester bond in an ATP-dependent manner. It transfers the activated ubiquitin molecule to a cysteine residue on the E2 enzyme to form an E2-ubiquitin thioester-linked intermediate. The E3 ligase facilitates transfer of the E2-ubiquitin moiety to the substrate protein *via* an amide bond between the carboxy terminus of ubiquitin and a lysine side chain of the substrate protein. The E3 ligase is substrate-specific, allowing repeated positioning of the distal end of ubiquitin molecule for ubiquitin chain assembly with high precision. The poly-ubiquitinated protein is then recognized for enzymatic degradation in the 26S proteasome (Powell, 2006; Schapira et al., 2019). Hence, the E3 reaction is critical for determining the turnover of specific proteins. There are 617 E3 ligases functionally annotated in the human genome (Li et al., 2008). It is known that F-box (FBXO) proteins are a key component of the Skp1-Cullin-F-box (SCF)-type ubiquitin ligase complex (SCF^FBXO^) and serve as sites for enzyme-substrate interaction (Kipreos and Pagano, 2000). FBXO proteins contain several functional domains such as the F-box domain, the LRRs, and the WD40 repeats for protein-protein interaction. Two muscle-specific FBXO proteins, FBXO-9 and FBXO-32 (also known as atrogin-1), have been found to be upregulated in diabetic vessels. They mediate BK-β1 protein ubiquitination in coronary arterial SMCs (Zhang et al., 2010a). The molecular basis of FBXO-32 and BK-β1 interaction was identified using site-directed mutagenesis and co-immunoprecipitation approaches, which showed that the PDZ-binding motif (ETSV) on BK-β1 is critical for FBXO-32-dependent ubiquitination (Zhang et al., 2010a). Deletion of the consensus sequence of the PDZ-binding motif in BK-β1 significantly decreases BK-β1 protein ubiquitination (Figure 4; Zhang et al., 2010a). Activation of FBXO proteins reduces BK-β1 expression, while knockdown of *FBXO* and proteasomal inhibition enhances BK-β1 levels, suggesting that accelerated UPS-mediated degradation of BK-β1 is an important mechanism of BK channel regulation in DM.

**Figure 5:**
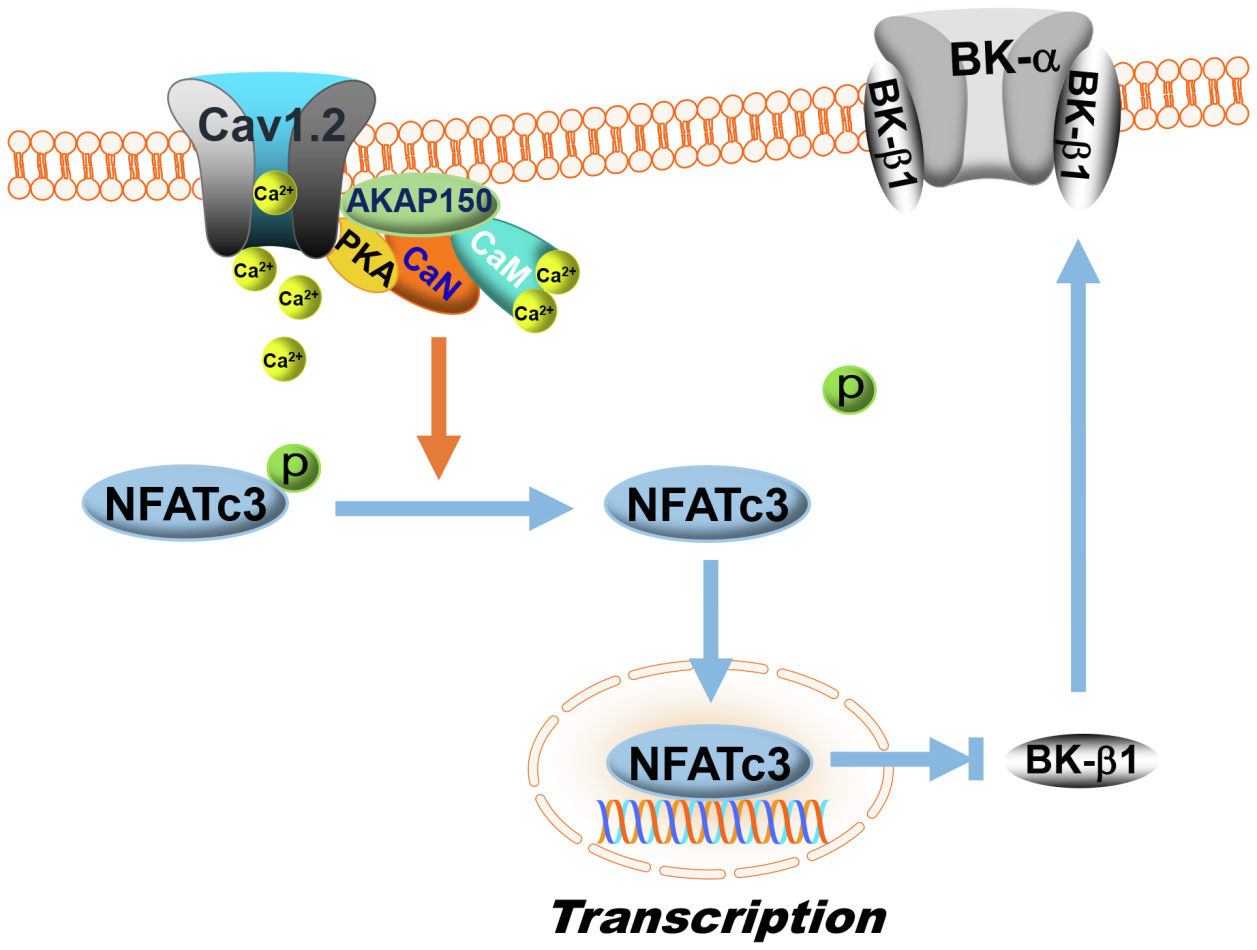
Regulation of BK-β1 expression by NFATc3 signaling. Calcineurin is a Ca^2+^/calmodulin (CaM)-activated phosphatase. In the membranes of vascular SMCs, AKAP150 proteins anchor calcineurin (CaN) with PKA and L-type Ca^2+^ channels (Cav1.2) to form dynamic Ca^2+^ signaling complexes. L-type Ca^2+^ channel activity is upregulated by PKA, which increases Ca^2+^ influx. Upon Ca^2+^ binding to calmodulin, calcineurin is activated, which then dephosphorylates NFATc3 and promotes NFATc3 nuclear translocation, inhibiting BK-β1 mRNA expression. In DM, the activity of the AKAP150-NFATc3 signaling pathway is upregulated, resulting in enhanced suppression of BK-β1 expression and impaired BK channel function in vascular SMCs. The symbol “p” represents protein phosphorylation.

The muscle RING-finger protein 1 (MuRF1) is another E3 ligase involved in UPS-dependent vascular BK-β1 degradation (Yi et al., 2014). Nuclear factor-κB (NF-κB) sites in the MuRF1 promoter are required for transcriptional activation, while FOXO sites are not (Wu et al., 2014). Overexpression of MuRF1 downregulates BK-β1 expression, impairs BK-β1-mediated BK channel activity, and reduces BK channel-induced vasodilation in mouse coronary arteries. We found that the N-terminus of BK-β1 and the coiled-coil region of MuRF1 are necessary for BK-β1 and MuRF1 interaction (Yi et al., 2014). Importantly, the protein expressions of FBXO-9, FBXO-32, and MuRF1 are unregulated in the arteries of STZ-induced T1DM animals and in primary human coronary arterial SMCs cultured with high glucose (Zhang et al., 2010a, 2020; Lu et al., 2012; Yi et al., 2014). Such upregulation of FBXO expression is mediated through the suppression of PI3K/AKT-dependent phosphorylation in FOXO-3a, thereby promoting FOXO-3a nuclear translocation and binding to the consensus sequence [GTAAA(C/T)A] in the promoter of *Fbxo* gene, activating its transcription (Furuyama et al., 2000). However, activation of MuRF1 is due to an increase of NF-κB-mediated *Trim63* (encoding MuRF1) transcription (Wu et al., 2014). In DM or hyperglycemia, the activity of AKT is reduced (Okon et al., 2005), while that of NF-κB is augmented (Narayanan et al., 2014), thereby promoting FBXO and MuRF1 expression (Figure 4). Indeed, inhibition of PKCβ activity by ruboxistaurin, NF-κB activity by TPCA-1, and proteasomal activity by MG132 downregulates BK-β1 ubiquitination, preserves BK-β1 expression, and improves BK channel function in coronary arterial SMCs (Zhang et al., 2010a; Lu et al., 2012; Yi et al., 2014).

BK-α protein expression is also regulated by lysosome and UPS degradation (Wang et al., 2013; Liu et al., 2014; Leo et al., 2015; Song et al., 2018). It has been found that the CRL4A and its substrate cereblon (CRBN) complex (CRL4A^CRBN^) serves as the ubiquitin ligase that interacts with the C-terminus of BK-α and induces BK-α protein degradation in neurons (Liu et al., 2014). A recent study reported that both CRBN and BK-α proteins were targeted by SCF^FBXO-7^ ubiquitin ligase complex for ubiquitination and proteolysis, controlling BK-α function and regulating the learning and memory processes in the brain (Song et al., 2018). However, the specific E3 ligase(s) responsible for BK-α protein ubiquitination in blood vessels is unknown, and how the BK-α-specific E3s are regulated in DM remains to be determined.

### Effects of Nuclear Factor Erythroid-2-Related Factor 2 Signaling on Vascular BK Channel Expression

Nuclear factor erythroid-2-related factor 2 (Nrf2) plays a critical role in the maintenance of intracellular redox homeostasis by regulating multiple downstream antioxidant enzymes and phase II detoxifying enzymes, which include NADPH dehydrogenase quinone 1 (NQO1), glutathione-disulfide reductase (GSR), glutathione translocase (GSTA), thioredoxin (TXN), thioredoxin reductase 1 (TXNRD1), heme oxygenase-1 (HO-1), SODs, CAT, and GPx (Gao and Mann, 2009; Chen et al., 2014). In addition, Nrf2 negatively regulates the expression of NOXs (McSweeney et al., 2016). The function of Nrf2 is principally regulated by the kelch-like ECH-association protein 1 (Keap1), which mediates Nrf2 ubiquitination and subsequent proteasomal degradation (Canning et al., 2015; Suzuki and Yamamoto, 2015). In the nuclei, Nrf2 binds to the promoters of antioxidant response elements (AREs) and electrophile response elements (EpREs) through interaction with the Nrf2-binding motif [TGA(G/C)xxxGC], where x represents any amino acid (Chorley et al., 2012). Both the *KCNMA1* and *KCNMB1* genes contain the consensus sequences of Nrf2-binding motifs in their promoter regions. Using promoter luciferase reporter assays, we confirmed that Nrf2 binds to the ARE of the *KCNMA1* promoter, but not to that of *KCNMB1* promoter. Mutation of the Nrf2-binding motif in the *KCNMA1* promoter abolishes the transcription response to Nrf2 (Sun et al., 2020). In addition, adenoviral expression of Nrf2 significantly augmented the mRNA levels of BK-α and BK-β1 in coronary arterial SMCs (Lu et al., 2017a; Sun et al., 2020). These results suggest that Nrf2 facilitates BK-α mRNA expression through activation of *KCNMA1* transcription, whereas the stimulatory effect of Nrf2 on BK-β1 mRNA expression is indirect and may be achieved by activating other transcription factor(s) or signaling mechanisms that upregulate *KCNMB1* transcription and expression in vascular SMCs.

Nuclear factor erythroid-2-related factor 2 deficiency has been implicated in diabetic complications including those associated with the heart (Tan et al., 2011; Bai et al., 2013), blood vessels (Ungvari et al., 2011; Miao et al., 2012; Li et al., 2017; Lu et al., 2017a), kidneys (Zheng et al., 2011; Cui et al., 2012), and the brain (Pu et al., 2018; Tarantini et al., 2018). The expression of Nrf2 and its downstream genes is slightly increased in the cardiovascular systems of STZ-induced T1DM mice at 2–3months after the onset of hyperglycemia, but then becomes significantly downregulated at 5–6months after the development of hyperglycemia (Tan et al., 2011; Miao et al., 2012; Bai et al., 2013), suggesting the burnout of an important redox protective mechanism in the advanced stages of DM. In db/db and HFD-induced diabetic mice 6months after the development of hyperglycemia, BK channel activity and BK channel-mediated vasodilation in coronary arteries are impaired, accompanied by a remarkable reduction in Nrf2 and its associated antioxidant enzymes (Li et al., 2017; Lu et al., 2017a). Nrf2 KO mice show excessive ROS production, as well as diminished BK channel expression and function in vascular SMCs (Ashino et al., 2013; Sun et al., 2020). Both mRNA and protein expression of BK-α are downregulated, whereas BK-β1 proteins but not mRNA levels are decreased in the arterial SMCs of Nrf2 KO mice, consistent with the notion that Nrf2 regulates BK-α *via* transcription, and BK-β1 through posttranscriptional mechanisms (Figure 4; Sun et al., 2020). Administration of dimethyl formamide (DMF, an FDA-approved Nrf2 activator) preserves BK channel protein expression, BK channel activity, and BK channel-mediated vasodilation in the coronary arteries of db/db and HDF-induced diabetic mice (Li et al., 2017; Lu et al., 2017a). Currently, Nrf2 activators such as DMF and sulforaphane (SFN) are being used in clinical trials for cardiovascular diseases and metabolic disorder (Yagishita et al., 2020), but it has not been administered for diabetic patients with coronary heart disease (Houghton, 2019). Whether the beneficial effects of Nrf2 activators observed in animal studies would translate into better outcomes in diabetic patients with cardiovascular complications needs to be determined.

### Effects of Calcineurin-Nuclear Factor of Activated T Cells Cytoplasmic 3 Isoform Pathway on BK-β1 Transcription

Nuclear factor of activated T cells cytoplasmic 3 isoform (NFATc3) belongs to the nuclear factor of activated T cells (NFAT) family of transcription factors that were originally discovered in resting T cells and is important in immune response (Rao et al., 1997). NFATc3 is also involved in the development of skeletal muscle and of the cardiovascular systems (Crabtree and Olson, 2002). The activity of NFATc3 is modulated by the Ca^2+^/calmodulin-dependent phosphatase, calcineurin. Elevation of the intracellular Ca^2+^ concentration activates calmodulin and promotes its binding to calcineurin, leading to calcineurin activation. Activated calcineurin dephosphorylates NFATc3, which in turn induces NFATc3 nuclear translocation. Calcineurin binds to the scaffolding protein A-kinase anchoring protein 150 (AKAP150), corresponding to AKAP79 in humans, which also anchors PKA and L-type Ca^2+^ channel to form a dynamic Ca^2+^ signaling complex (Oliveria et al., 2007). AKAP79/150 strongly suppresses PKA-mediated L-type Ca^2+^ channel phosphorylation and is required for the activation of NFAT by local Ca^2+^ influx through L-type channels (Oliveria et al., 2007).

Nuclear factor of activated T cells share a conserved DNA-binding domain that specifically binds to the DNA core sequence [(A/T)GGAAA] at the promoter region of target genes, activating gene transcription (Rao et al., 1997). Human and mouse *KCNMA1* and *KCNMB1* contain at least one NFAT-binding motif in their promoters. Inhibition of vascular BK channels by NFATc3 has been reported, while upregulation of NFATc3 expression by Ang II results in decreased BK channel activity in mouse arteries due to the downregulation of BK-β1 mRNA expression (Nieves-Cintron et al., 2007). The effects of NFATc3 on BK channel activity and BK-β1 mRNA expression are abolished by calcineurin inhibitors, FK506 and cyclosporin A, in the presence of Ang II, a finding that has been confirmed in NFATc3 KO mice (Nieves-Cintron et al., 2007). AKAP150 also participates in NFATc3-mediated BK channel downregulation in HFD-induced diabetic mice (Figure 5; Nystoriak et al., 2014). In HFD-induced diabetic mice, the activity of the AKAP150-NFATc3 signaling pathway is upregulated, contributing to impaired BK channel function with reduced BK-β1 expression and increased vascular tone in the mesenteric arteries. However, in AKAP150 KO mice with HFD consumption, the deleterious effects of HFD on BK channels are not observed (Nystoriak et al., 2014). Recently, *in vivo* administration of a NFATc3 inhibitor (A285222, Abbott Labs) in Akita T1DM mice is found to improve vascular endothelial function, enhance eNOS activity and NO production, reduce endothelin-1 secretion, lower blood pressure, and improve survival (Garcia-Vaz et al., 2020). The beneficial effects of NFATc3 inhibitors on coronary BK channel function in DM warrant further investigation.

### Arachidonic Acid and Its Metabolites on BK Channel Regulation

Arachidonic acid (AA), a polyunsaturated omega-6 fatty acid, is abundant in normal human diet and in membrane phospholipids. It is an important precursor to a wide range of bioactive mediators and eicosanoids that regulate a multitude of essential functions in the body (Tallima and El Ridi, 2018). AA is metabolized by three major enzyme systems: It is converted by 12-lipoxygenase (12-LOX) into leukotrienes and 12-hydroxyeicosatetraenoic acid (12-HETE), by cytochrome P-450 (CYP-450) epoxygenase into epoxyeicosatrienoic acids (EETs), and by cyclooxygenases (COX) into prostaglandins, including PGI_2_ and thromboxane A2 (TXA_2_; Brash, 2001; Vila, 2004). Additionally, AA can be metabolized by CYP-450 omega-hydroxylase to produce 20-hydroxyeicosatetraenoic acid (20-HETE).

Arachidonic acid (Lu et al., 2005; Kur et al., 2014; Martin et al., 2014, 2021) and its metabolites (EETs, PGI_2_, 12-HETE, and 20-HETE; Li and Campbell, 1997; Yamaki et al., 2001; Zhang et al., 2001; Zink et al., 2001; Lauterbach et al., 2002; Morin et al., 2007) are known to activate vascular BK channels and promote vasodilation through endothelium-dependent hyperpolarization mechanisms. Direct exposure to 10μM AA robustly increases BK channel activity in inside-out excised patches from human umbilical arterial SMCs, suggesting activation of BK channels directly by AA (Martin et al., 2021). Extracellular application of AA results in BK channel activation and hyperpolarization of resting membrane potentials in vascular SMCs (Kur et al., 2014; Martin et al., 2021). These changes can be blocked by LOX, CYP, and COX inhibitors, suggesting that AA metabolites affect BK channels. The effects of AA on BK channels require the presence of BK-β1 (Sun et al., 2007; Martin et al., 2021).

The activation of vascular BK channels by PGI_2_ is associated with cAMP-dependent, PKA-mediated phosphorylation. EETs and their metabolites dihydroxyeicosatrienoic acids (DHETs) are also potent BK channel activators and vasodilators, including the human coronary microvessels and internal mammary arteries (Quilley et al., 1997; Archer et al., 2003; Feletou and Vanhoutte, 2006; Larsen et al., 2006). Several different mechanisms of EET- and DHET-mediated BK channel activation have been proposed, including direct activation (Wu et al., 2000; Lu et al., 2001), ADP-ribosylation of Gsα (Fukao et al., 2001; Li et al., 2002), and stimulation of PKA-mediated phosphorylation (Dimitropoulou et al., 2007; Imig et al., 2008). However, AA-induced vasodilation of coronary arterioles *via* BK channel activity is impaired in high glucose conditions and DM (Lu et al., 2005; Zhou et al., 2005, 2006; Yousif and Benter, 2007; Tsai et al., 2011). PGI_2_ and EET levels are decreased in patients with cardiovascular diseases (Theken et al., 2012; Mokhtar et al., 2013; Schuck et al., 2013) and DM (Lane et al., 1982; Kazama et al., 1987; Migdalis et al., 2001; Duflot et al., 2019). As a result of these findings, AA metabolites and analogues have been developed as potential therapeutic agents for cardiovascular diseases and diabetic vascular complications (Campbell et al., 2017; Wang et al., 2021).

## Future Directions in Diabetic BK Channel Research

Studies of the regulation of BK channel function and expression have greatly advanced our understanding on the role of BK channels in diabetic cardiovascular complications. DM involves a plethora of signaling abnormalities including those pertaining to insulin, ROS generation, Ang II signaling, and Ca^2+^ regulation. Thus, it is not surprising that DM affects vascular BK channel expression and function in many different ways, including transcription, translation, post-translation, surface trafficking, and channel degradation. Whether surface trafficking dysregulation of BK channel subunits contributes to BK channelopathy of the vascular SMCs in DM is unknown. Moreover, BK channels do not exist as isolated proteins but are assembled in membrane microdomains of vascular ECs and SMCs. Studies of BK channel organization by scaffolding proteins in close proximity with receptors, enzymes, and Ca^2+^ sources in blood vessels will provide further insights into BK channel physiology and into the molecular mechanisms underlying BK channelopathy in DM. In addition, our knowledge on BK-γ1 in diabetic BK channel dysregulation is very limited. Little is known about the regulation of vascular BK-γ1 expression and function in hyperglycemia and DM. Since the results of BK channel pathology from diabetic animal models are diverse, it is critical to study vascular BK channel biology and dysfunction using human tissues, which serve as the gold standard for diabetic BK channel research.

Ca^2+^-activated K^+^ channels are important regulators of vascular physiology and are critical determinants coronary circulation and cardioprotection. Preservation of BK channel expression and activities protects vascular function in DM. Hence, a better understanding of BK channelopathy and prevention of BK channel abnormalities in DM may lead to better vascular therapeutics and care for patients with DM.

## Author Contributions

TL and HL wrote the manuscript and critically reviewed the final version of the manuscript. All authors contributed to the article and approved the submitted version.

## Funding

This work was supported by grants from the National Institute of Health (RO1 HL-080118 and RO1 HL-074180), American Diabetes Association (ADA-JFA-07-39, ADA 1-12-BS-119, ADA 1-16-IBS-195, and ADA 1-18-IBS-210), and the Mayo Clinic.

## Conflict of Interest

The authors declare that the research was conducted in the absence of any commercial or financial relationships that could be construed as a potential conflict of interest.

## Publisher’s Note

All claims expressed in this article are solely those of the authors and do not necessarily represent those of their affiliated organizations, or those of the publisher, the editors and the reviewers. Any product that may be evaluated in this article, or claim that may be made by its manufacturer, is not guaranteed or endorsed by the publisher.

## Footnotes

^1^ https://www.who.int/health-topics/diabetes#tab=tab_1
